# Rescue Cervical Cerclage for Previable Birth Prevention: A Comprehensive Review of Indications, Techniques, and Outcomes

**DOI:** 10.7759/cureus.68619

**Published:** 2024-09-04

**Authors:** Prachi A Ughade, Deepti Shrivastava, Kamlesh Chaudhari

**Affiliations:** 1 Obstetrics and Gynaecology, Jawaharlal Nehru Medical College, Datta Meghe Institute of Higher Education and Research, Wardha, IND

**Keywords:** preterm birth prevention, pregnancy outcomes, rescue cerclage, cervical incompetence, previable birth, cervical cerclage

## Abstract

Cervical cerclage is a surgical intervention aimed at preventing previable birth by providing mechanical support to the cervix through the placement of a suture. This procedure is primarily indicated for cases of cervical incompetence, where the cervix is prone to premature dilation and can lead to early miscarriage or preterm birth. This review seeks to comprehensively analyze rescue cervical cerclage (RCC), focusing on its indications, techniques, and outcomes. The goal is to offer an in-depth understanding of its effectiveness in preventing previable birth and to guide clinical decision-making in managing pregnancies at risk of premature delivery. A thorough literature review examined recent studies and clinical data on RCC. Key aspects evaluated include the criteria for intervention, various surgical techniques employed, and the associated maternal and fetal outcomes. Comparative analysis was also performed with other preventive measures, such as progesterone therapy and bed rest. RCC has demonstrated efficacy in reducing the incidence of previable births, particularly when performed in cases of identified cervical incompetence or shortening. The review highlights variations in the technique, such as McDonald and Shirodkar cerclage, and discusses their relative success rates and potential complications. The procedure is associated with improved pregnancy outcomes and reduced risk of previable birth, though it carries some risks, including infection and cervical laceration. RCC remains a valuable intervention for preventing previable births in selected patients. This review underscores its importance in managing pregnancies at risk due to cervical incompetence and provides a detailed evaluation of its indications, techniques, and outcomes. Future research should optimize criteria for cerclage placement and explore novel strategies to enhance its effectiveness and safety.

## Introduction and background

Cervical cerclage is a surgical procedure designed to prevent preterm birth (PTB) by providing additional support to the cervix through the placement of a stitch or suture. This intervention is commonly used in cases of cervical incompetence, a condition where the cervix fails to maintain its closure during pregnancy, leading to premature dilation and a heightened risk of miscarriage or preterm delivery [1]. By reinforcing the cervical structure, cerclage aims to reduce the likelihood of premature cervical opening and extend the duration of the pregnancy, improving the chances for fetal viability and survival [2].

The importance of cervical cerclage in preventing previable birth cannot be overstated. Previable birth, typically defined as delivery occurring before 24 weeks of gestation, poses significant risks to both maternal and fetal health. Infants born at this stage face extremely high rates of morbidity and mortality due to underdeveloped organs and systems [3]. By addressing the issue of cervical incompetence early and effectively, rescue cervical cerclage (RCC) can play a crucial role in reducing the incidence of previable births, thereby improving neonatal outcomes and enhancing the prospects for fetal development and survival [4].

The primary objective of this review is to offer a comprehensive analysis of RCC as a strategy for preventing previable birth. This review aims to critically evaluate the procedure's indications, the techniques employed, and the associated outcomes. By synthesizing current evidence and clinical experiences, the review seeks to provide a detailed understanding of the effectiveness and practicality of cervical cerclage in managing pregnancies at risk of previable birth. This review's scope extends to examining the criteria that justify the use of RCC, including the specific clinical indications and timing of the intervention.

## Review

Indications for RCC

RCC is a surgical procedure designed to prevent previable birth in women with cervical insufficiency. This intervention is indicated based on general and specific criteria and considerations related to the timing and criteria for the procedure [3]. General indications for RCC include a history of cervical incompetence and previous PTBs. Women who have previously experienced cervical insufficiency - characterized by painless cervical dilation leading to losses in the second trimester - are at higher risk for similar complications in subsequent pregnancies. Additionally, a history of PTBs, especially those attributed to cervical insufficiency, is a significant indicator for considering cerclage. These factors underscore the importance of a comprehensive obstetric history in identifying candidates who may benefit from this intervention [1]. Specific indications for rescue cerclage often arise during pregnancy and include symptoms such as cervical shortening and funnelling. These symptoms can be identified through physical examinations or ultrasound assessments. Risk factors that increase the likelihood of cervical insufficiency include multiple pregnancies (e.g., twins or higher-order multiples), uterine anomalies (e.g., a bicornuate uterus), and a history of cervical surgeries or trauma. Recognizing these specific indications is crucial for timely intervention [5]. The timing of RCC is critical to its success. Generally, the procedure is performed after 24 weeks of gestation when cervical changes become apparent [6]. However, guidelines suggest that intervention may be beneficial between 16 and 27 weeks of gestation, particularly when the cervix is dilated. Still, there are no signs of active labour, infection, or significant bleeding. Clinical criteria for intervention include cervical dilation greater than 2 cm in the absence of labour or infection. The overall clinical context, including the potential to extend the pregnancy to a viable gestational age, is essential in the decision-making process [7]. The descriptions of RCC are detailed in Table 1.

**Table 1:** Indications for Rescue Cervical Cerclage

Category	Indications
General Indications	History of cervical incompetence (painless cervical dilation leading to second-trimester losses)
	Previous preterm births attributed to cervical insufficiency
Specific Indications	Cervical shortening and funneling detected via ultrasound
	Presence of risk factors such as multiple pregnancies (e.g., twins)
	Uterine anomalies (e.g., bicornuate uterus)
	History of cervical surgeries or trauma
Timing of Intervention	Typically performed between 16 and 27 weeks of gestation
	Indicated when cervical dilation is greater than 2 cm without signs of labour, infection, or bleeding
	Consideration of the overall clinical picture, including the potential to prolong pregnancy

Techniques of RCC

RCC is a surgical procedure designed to prevent previable birth in women with cervical insufficiency. This intervention can be performed using various techniques, with the McDonald and Shirodkar cerclages being the most prevalent. The McDonald cerclage involves placing a purse-string suture around the cervix, typically between 16 and 18 weeks of pregnancy [1]. This method is relatively straightforward and allows for easy suture removal around 37 weeks or earlier if necessary. In contrast, the Shirodkar cerclage involves sutures passing through the cervical walls, which remain hidden, potentially reducing the risk of infection. This technique is more complex and is often chosen for cases requiring a more permanent solution, as it may necessitate cesarean delivery. Additionally, transabdominal cerclage may be employed when the cervix is too short or when previous vaginal cerclage has failed [8]. The procedure is generally performed after 24 weeks of gestation when the cervix has begun to dilate. The cerclage involves suturing the cervix to provide structural support, thereby maintaining cervical length and preserving the integrity of the mucus plug, which acts as a barrier against infection [9]. The surgery is usually carried out under spinal anaesthesia to minimize discomfort. Absolute contraindications for this procedure include active labour, significant bleeding, placental abruption, premature rupture of membranes (PROM), and chorioamnionitis. Relative contraindications may include cases with prolapsed membranes or light spotting [10]. Postoperative care is crucial following RCC due to the higher risk of complications compared to elective cerclage. Complications can occur in up to 58% of cases and may include PROM, chorioamnionitis, and, in rare instances, fistula formation. Close monitoring for signs of infection or preterm labour is essential. Tocolytics may be administered to suppress contractions, and prophylactic antibiotics are often given to reduce the risk of infection. Given the nature of the procedure, delivery is usually planned via cesarean section to avoid trauma to the cervix [11]. Techniques for RCC are detailed in Table 2.

**Table 2:** Techniques for Rescue Cervical Cerclage

Technique	Description	Indications	Advantages	Disadvantages
McDonald Cerclage	Placement of a purse-string suture around the cervix, typically between 16 and 18 weeks of pregnancy.	General cases of cervical incompetence.	Simple procedure; suture easily removed near term.	Less secure in some cases; may require removal if complications arise.
Shirodkar Cerclage	Sutures passed through the cervical walls, with sutures not exposed, providing more permanent support.	Cases needing a more permanent solution; higher risk pregnancies.	Reduced risk of infection; more secure support.	More complex procedures may necessitate cesarean delivery.
Transabdominal Cerclage	Sutures are placed around the cervix through the abdomen, typically when the cervix is too short or previous cerclage has failed.	Short or incompetent cervix; previous cerclage failure.	Suitable for very short cervixes; can be left in place for future pregnancies.	Invasive procedure; requires abdominal surgery, often requiring cesarean delivery.
Laparoscopic Cerclage	Placement of cerclage via a minimally invasive laparoscopic approach.	Cases requiring less invasive procedure or failed previous cerclage.	Minimally invasive; reduced recovery time.	It requires specialized surgical skills, which may not be widely available.
Biodegradable Cerclage	Use biodegradable materials to support, avoiding the need for suture removal.	Research and experimental cases.	There is no need for suture removal; there is a potentially lower infection risk.	Still under research; long-term outcomes not well established.

Outcomes of RCC

RCC is a surgical procedure designed to extend pregnancy in cases of cervical insufficiency, particularly when the cervix is already dilated. The outcomes of this intervention can be categorized into efficacy, complications, and long-term effects [12]. Regarding efficacy, research indicates that rescue cerclage can significantly extend gestation and enhance neonatal outcomes compared to expectant management. For example, a systematic review found that emergency cervical cerclage improved overall survival by 43%, fetal survival by 17%, and neonatal survival by 22% compared to conservative treatments. Despite these improvements, the average gestational age at delivery following cerclage is around 30.6 weeks, and the incidence of extremely preterm delivery (before 24 weeks) remains high at approximately 23% [12]. Successful cerclage placement is associated with better perinatal outcomes, including reduced neonatal morbidity and mortality rates. Factors influencing success include earlier gestational age at diagnosis, negative AmniSure test results, and smaller cervical dilatation at the time of cerclage [12]. Maternal risks associated with rescue cerclage include infections, cervical lacerations, and PROM. The procedure may also lead to complications such as uterine contractions, which can result in early delivery or other adverse maternal outcomes. Fetal risks include PTB and fetal distress. Despite the intervention, the likelihood of PTB remains significant, with many studies reporting high rates of preterm deliveries [13]. The long-term implications of rescue cerclage on future pregnancies are not well-defined. Some studies suggest that women who have undergone cerclage may experience changes in cervical integrity in subsequent pregnancies, potentially increasing their risk of cervical insufficiency again [14]. The psychological and physical impacts of rescue cerclage can affect the quality of life for women, particularly due to the stress of high-risk pregnancies and potential complications. Long-term follow-up studies are needed to evaluate the overall quality of life and reproductive health in women following cerclage [15].

Comparison with other interventions

Non-surgical alternatives for managing cervical insufficiency primarily include bed rest and progesterone therapy. Traditionally, bed rest has been recommended for women at risk of PTB due to cervical insufficiency. However, recent studies have shown that bed rest alone is not effective in preventing PTB and may pose risks such as blood clots and muscle weakening [16]. Research indicates that women who received emergency cervical cerclage had significantly better outcomes compared to those who relied solely on bed rest. Specifically, cerclage recipients experienced longer gestational ages at delivery and lower rates of preterm membrane rupture, suggesting that bed rest alone is insufficient [15]. Progesterone supplementation is another non-surgical alternative that has gained attention for its potential to prevent PTB, particularly in women with a history of PTB or cervical insufficiency. While progesterone has shown promise in certain populations, its effectiveness compared to cerclage is still under investigation [17]. Some studies suggest that combining progesterone with cerclage may improve outcomes, but further research is needed to establish its comparative effectiveness. This combined approach might offer a more comprehensive strategy for managing at-risk pregnancies [18,19]. Combining cerclage with other treatments has also been explored to enhance pregnancy outcomes for women with cervical insufficiency. When used in conjunction with bed rest, cerclage has been shown to significantly reduce preterm delivery rates and neonatal morbidity compared to bed rest alone. This suggests that while bed rest may not be effective as a standalone intervention, it could still be beneficial as part of a multifaceted treatment plan. The effectiveness of combining cerclage with progesterone therapy remains under investigation, with some studies indicating potential benefits [20]. Comparative studies of cerclage versus non-surgical alternatives, such as bed rest and progesterone therapy, indicate that cerclage is generally more effective in prolonging pregnancy and improving neonatal outcomes for cases of cervical insufficiency. The choice of intervention often depends on individual patient factors, including cervical length and obstetric history, underscoring the need for personalized treatment plans. As research evolves, refining these approaches will be crucial in establishing optimal treatment protocols for women at risk of previable birth [21]. A comparison of RCC with other interventions for preventing previable birth is detailed in Table 3.

**Table 3:** Comparison of Rescue Cervical Cerclage With Other Interventions for Preventing Previable Birth PROM: premature rupture of membranes

Intervention	Description	Effectiveness	Advantages	Disadvantages
Rescue Cervical Cerclage	Surgical placement of a suture around the cervix to provide mechanical support and prevent premature dilation.	Effective in prolonging pregnancy and improving neonatal outcomes in cases of cervical insufficiency.	Direct mechanical support to the cervix can significantly reduce preterm birth rates in indicated cases.	Invasive procedures are associated with risks such as infection, PROM, and cervical lacerations.
Bed Rest	Reducing physical activity and remaining in bed to minimize pressure on the cervix.	Limited effectiveness in preventing preterm birth may not provide significant benefits when used alone.	Non-invasive; easy to implement.	It may increase risks of blood clots, muscle weakening, and other complications; it lacks strong evidence of efficacy.
Progesterone Therapy	Administration of progesterone to help maintain pregnancy and reduce the risk of preterm birth.	Shows promise in certain populations, particularly with a history of preterm birth or cervical shortening.	Non-invasive; can be used in combination with other interventions such as cerclage.	Effectiveness is still under investigation; it may not be sufficient alone for severe cases of cervical insufficiency.
Combination: Cerclage + Bed Rest	Use cervical cerclage in conjunction with bed rest to provide mechanical support and reduce physical strain.	More effective than bed rest alone in reducing preterm delivery rates and neonatal morbidity.	It combines mechanical and non-invasive support and may offer better outcomes than bed rest alone.	Bed rest components may still pose risks; overall effectiveness depends on patient-specific factors.
Combination: Cerclage + Progesterone	Combining cervical cerclage with progesterone therapy to enhance the prevention of preterm birth.	Some studies suggest potential benefits, though more research is needed to confirm comparative effectiveness.	Combining mechanical support with hormonal therapy may improve outcomes in high-risk pregnancies.	Lack of large-scale studies; potential risks and benefits need further exploration.

Controversies and challenges

RCC is surrounded by several controversies and challenges, particularly concerning its indications and technical and ethical considerations. One major area of debate involves the differing guidelines and recommendations for RCC [3]. Various professional organizations, such as the Society of Obstetrics and Gynecology of Canada (SOGC) and the National Institute for Health and Care Excellence (NICE), provide conflicting criteria for when RCC should be considered. For instance, the SOGC guidelines suggest that RCC may be appropriate for women with cervical dilation of less than 4 cm before 24 weeks of gestation [22]. In contrast, NICE guidelines recommend consideration for women between 16 and 27 weeks with a dilated cervix and exposed, unruptured fetal membranes. This discrepancy in recommendations arises from the absence of large-scale randomized controlled trials, leading to reliance on smaller studies and expert opinions. Consequently, this variability can cause confusion among practitioners and patients regarding the appropriateness of the procedure in various clinical contexts [23]. In addition to the debate on indications, significant technical and ethical issues are associated with RCC. The execution of RCC varies widely among practitioners, influenced by individual clinical experiences and institutional protocols. This variability can lead to differences in outcomes, complicating the establishment of standardized practices. The lack of consensus on the optimal timing and patient selection criteria further exacerbates this issue, making it difficult to determine best practices across different healthcare settings. As a result, inconsistencies in how RCC is performed can impact the overall effectiveness and safety of the procedure [24]. Ethical dilemmas also emerge in the decision-making process for RCC, particularly regarding informed consent and the potential risks involved. The procedure carries risks such as infection and preterm PROM, which must be communicated to patients. Additionally, ethical considerations include the implications of prolonging a pregnancy that may ultimately result in a nonviable outcome, raising questions about the quality of life for the infant and the emotional impact on the family. These complexities necessitate careful deliberation and individualized patient counselling to navigate the challenges associated with this procedure [25]. Controversies and challenges in RCC are detailed in Table 4.

**Table 4:** Controversies and Challenges in Rescue Cervical Cerclage SOGC: Society of Obstetrics and Gynecology of Canada; NICE: National Institute for Health and Care Excellence; PROM: premature rupture of membranes

Category	Controversy/Challenge	Description	Implications
Indications	Variability in Guidelines	Different professional organizations (e.g., SOGC, NICE) have varying criteria for considering rescue cervical cerclage.	This inconsistency can lead to confusion among practitioners and patients, making it challenging to determine the appropriateness of the procedure.
Timing of Intervention	Optimal Timing Unclear	The ideal time to perform rescue cerclage remains debated, with guidelines differing on the recommended gestational age and cervical dilation.	Inconsistent timing recommendations can affect the success of the procedure and outcomes for both mother and fetus.
Technical Execution	Variability in Surgical Techniques	Surgeons may employ techniques based on personal experience, institutional protocols, or patient anatomy.	This variability can result in differing outcomes, complicating efforts to establish standardized practices.
Ethical Considerations	Informed Consent and Risk Communication	The procedure carries risks such as infection and PROM, which must be communicated to patients.	Ethical dilemmas arise when balancing the risks of the procedure against the potential benefits, especially in high-risk pregnancies.
Long-Term Outcomes	Lack of Long-Term Data	There is limited data on the long-term effects of rescue cerclage on future pregnancies and maternal health.	The absence of comprehensive long-term studies makes it difficult to fully understand the procedure's impact on reproductive health and quality of life.
Patient Selection	Difficulty in Identifying Ideal Candidates	There is no universally accepted criteria for selecting patients most likely to benefit from rescue cerclage.	Inadequate selection criteria can lead to suboptimal outcomes through unnecessary interventions or missed opportunities for those who could benefit.
Effectiveness in Diverse Populations	Limited Research in Diverse Populations	Most studies have not thoroughly explored how ethnicity, socioeconomic status, and comorbidities influence outcomes.	This gap in research may lead to inequities in care and less effective interventions for certain populations.

Future directions and research

RCC for Previable Birth Prevention

RCC is a debated procedure used to prevent previable birth in cases of cervical insufficiency. This technique involves surgically placing a stitch around the cervix to provide additional support and prevent preterm delivery. Typically, rescue cerclage is considered for pregnancies between 16 and 26 weeks of gestation, particularly when the cervix has begun to dilate and bulging membranes are present. It is often used in women with a history of PTB or second-trimester loss suggestive of cervical incompetence [3]. The procedure usually involves placing a suture around the cervix through the vaginal route. Amniocentesis may be performed before the cerclage to rule out infection. Antibiotics and tocolytics are frequently administered as part of the protocol. While rescue cerclage can extend pregnancy to a viable gestational age in some cases, with reported survival rates ranging from 48% to 68%, it carries risks such as infection and preterm PROM. Therefore, the benefits must be carefully weighed against each case's potential risks [26].

Future Directions and Research in RCC

**Figure 1:**
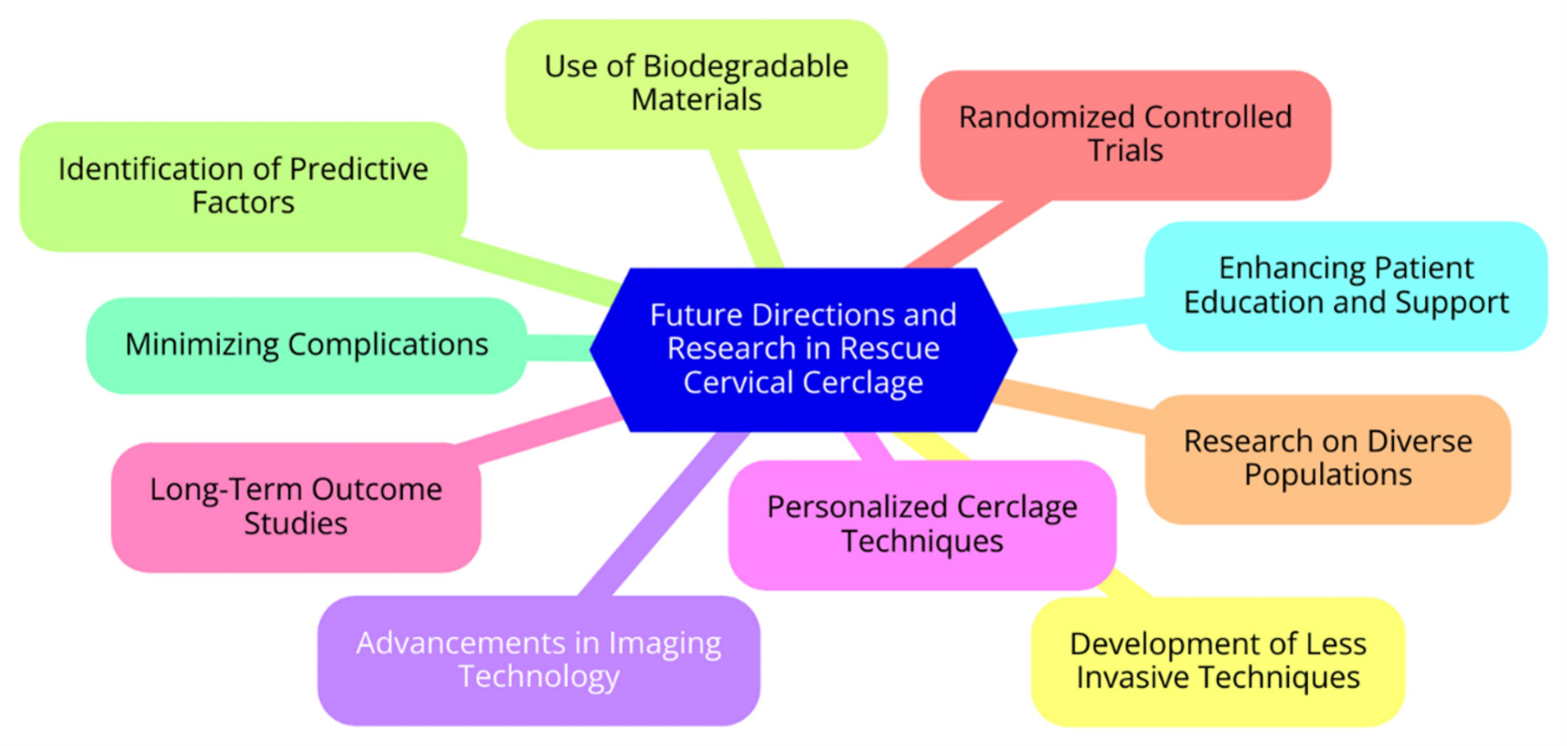
Future Directions and Research in Rescue Cervical Cerclage Image Credit: Dr. Prachi Ughade

As our understanding of cervical insufficiency and PTB continues to advance, so does research and development surrounding RCC. There is increasing interest in exploring less invasive techniques for cerclage placement, such as laparoscopic or transabdominal approaches, which may reduce complications associated with traditional vaginal cerclage. The use of biodegradable materials for cerclage is also under investigation, with the potential to minimize the need for suture removal and reduce infection risk. Ongoing research aims to evaluate the effectiveness and safety of these materials [27]. Advancements in imaging technology, such as ultrasound guidance, can enhance the precision of cerclage placement, potentially leading to improved outcomes and reduced complication rates. Personalizing cerclage techniques based on individual patient anatomy and risk factors - such as assessing cervical length and uterine tone more accurately before selecting the type of cerclage - could further enhance efficacy [28]. Long-term studies are needed to assess the immediate outcomes and the lasting effects of rescue cerclage on maternal and neonatal health. More randomized controlled trials are called to compare rescue cerclage to other interventions or conservative management strategies to establish clearer guidelines and best practices. Research should include diverse populations to understand how factors such as ethnicity, socioeconomic status, and comorbidities influence the effectiveness of cerclage [29]. Identifying specific predictive factors to determine which patients most likely benefit from rescue cerclage could lead to more targeted interventions. Additionally, investigating strategies to minimize complications, such as infection and preterm PROM, is crucial for improving overall outcomes. Enhancing patient education and psychological support for those undergoing cerclage can improve adherence to post-operative care and overall patient satisfaction [30]. Future research and advancements in RCC techniques hold promise for improving outcomes for women at risk of previable birth. Addressing existing gaps in knowledge and focusing on innovative approaches will be key to enhancing the safety and efficacy of this critical intervention. Continued collaboration among researchers, clinicians, and patients will be essential in shaping the future of cervical cerclage practices [4]. Future directions and research in RCC are illustrated in Figure 1.

## Conclusions

In conclusion, RCC represents a valuable intervention for preventing previable birth in pregnancies complicated by cervical incompetence. This review has highlighted the critical role of cerclage in extending pregnancy duration and improving neonatal outcomes by reinforcing cervical support. Despite its benefits, the procedure is not without risks, and careful consideration of indications, surgical techniques, and potential complications is essential for optimal results. This review aims to guide clinicians in making informed decisions and enhance patient management strategies by evaluating current evidence and practices. The ongoing advancements in techniques and understanding of cervical cerclage underscore the need for continued research to refine the procedure and address existing challenges. Ultimately, improving the effectiveness and safety of RCC can significantly contribute to reducing the incidence of previable births and advancing prenatal care.
